# Integrating mental health into emergency preparedness and response: lessons learned from Covid-19

**DOI:** 10.1093/eurpub/ckac131.055

**Published:** 2022-10-25

**Authors:** MA Alkasaby, F Baingana, WK Bosu, M Abdulaziz, R Mwaisaka, A Kakunze, N Keita, K Saeed, J Eaton, I Walker

**Affiliations:** 1 Centre for Global Mental Health, LSHTM, London, UK; 2 UK Public Health Rapid Support Team, UK Health Security Agency, LSHTM, London, UK; 3 Regional Advisor for Mental Health, WHO Regional Office for Africa, Brazzaville, Congo (Brazzaville); 4 West African Health Organisation, Bobo-Dioulasso, Burkina Faso; 5 Africa Centres for Disease Control and Prevention, Addis Ababa, Ethiopia; 6 East, Central and Southern African Health Community, Arusha, Tanzania; 7 Regional Advisor for Mental Health, WHO Regional Office for Eastern Mediterranean, Cairo, Egypt; 8 CBM Global Disability and Inclusion, Amstelveen, Netherlands; 9 Office for Health Improvement and Disparities, Department of Health and Social Care, London, UK

## Abstract

**Introduction:**

The COVID-19 crisis has disrupted health systems all over the world. In a survey by the WHO, 93% of the countries reported disruption in their mental health services. This research assessed the extent to which mental health was included in the national response to the COVID-19 pandemic in African countries. It also explored barriers and enablers to mental health integration into the COVID-19 response. Lessons learned from COVID-19 can help improve the response to future public health emergencies.

**Methods:**

A web-based survey was sent to mental health focal points in 55 African countries. The survey assessed the perceived degree of implementation of the Inter-Agency Standing Committee (IASC) “14 Globally Recommended Activities” for mental health response to COVID-19. This was followed by in-depth interviews to explore barriers and enablers to mental health integration into the COVID-19 response.

**Results:**

Responses were received from 28 countries. Lack of political will, poor funding, limited human resources, and weak pre-existing mental health systems were the key challenges in addressing mental health needs during COVID-19. Participants highlighted the need to capitalize on the increased attention to mental health during COVID-19 to support its integration into the emergency preparedness and response plans and strengthen health systems in the longer term. They have also stressed the importance of sustaining and strengthening the new partnerships and service delivery models that emerged during the COVID-19 pandemic.

**Conclusions:**

The number of recommended mental health activities implemented during the COVID-19 pandemic varied considerably across African countries. Several factors limit mental health integration into emergency response. However, there are signs of optimism, as mental health gained some attention during COVID-19, which can be built on to integrate mental health into emergency response and strengthen health systems in the long term.

**Key messages:**

• Capitalize on the increased attention to mental health during COVID-19 to support its integration into the emergency preparedness and response plans and strengthen health systems in the long term.

• Sustain and strengthen the new partnerships and service delivery models that emerged during the COVID-19 pandemic.

